# Quantitative evaluation of activity of thyroid-associated Ophthalmopathy using short-tau inversion recovery (STIR) sequence

**DOI:** 10.1186/s12902-021-00895-3

**Published:** 2021-11-13

**Authors:** Qian Ge, Xiaohui Zhang, Lu Wang, Yao Fan, Qian Huang, Ning Yao, Jian Long, Chun Liu

**Affiliations:** 1 Department of Endocrinology, the First Affiliated Hospital of Chongqing Medical University, NO.1, Friendship road, Yuzhong District, Chongqing, 400016 China; 2 Department of Medical imaging, the First Affiliated Hospital of Chongqing Medical University, Chongqing, 400016 China; 3 Chongqing center for disease control and prevention, Chongqing, 400042 China

**Keywords:** Thyroid-associated ophthalmopathy, Magnetic resonance imaging, Extraocular muscle, Short-tau inversion recovery

## Abstract

**Objective:**

Quantitatively staging TAO using MRI remains limited. Our study aims to identify the cut-off signal intensity value for staging TAO using STIR sequence scan.

**Methods:**

Between June 2018 and July 2020, a number of 51 patients with TAO (102 eyes) and 19 volunteer controls (38 eyes) were recruited. The clinical and biochemical parameters were measured in each patient. Disease activity was diagnosed based on the Clinical Activity Score (CAS). The signal intensities of extraocular muscles were scanned using short-tau inversion recovery (STIR) sequences from MRI.

**Results:**

Compared to the inactive TAO patients and the controls, the signal intensity ratios (SIRs) of the superior rectus, inferior rectus, medial rectus, lateral rectus on STIR images were significantly increased in the active TAO patients. After adjustment for age and smokers, the SIRs of four extraocular muscles showed strong associations with CAS. By receiver operator characteristic (ROC) curve analysis, all four muscle SIRs demonstrated good efficiency for predicting disease activity [area under curve (AUC) 0.75–0.83, all *P* < 0.01]. The identified cut-off SIR values were further validated in a new group of TAO patients (30 eyes) enrolled between September 2020 and January 2021. The cut-off SIR value of > 2.9 in the inferior rectus showed optimal diagnostic value for staging the active TAO.

**Conclusions:**

the signal intensity of extraocular muscles on STIR sequence was a good predictor for TAO activity. A cut-off SIR value of > 2.9 in the inferior rectus could be applied to evaluate the active stage of TAO.

**Supplementary Information:**

The online version contains supplementary material available at 10.1186/s12902-021-00895-3.

## Introduction

Thyroid-associated ophthalmopathy (TAO) is a chronic inflammatory autoimmune disease of periorbital tissue [1–3]. The course of the disease can be divided into two stages: active inflammation and inactive fibrosis. The active stage is characterized by lymphocytic infiltration, edema in the extraocular muscles, and orbital fat expansion. In contrast, the inactive stage is featured with fibrosis and fatty degeneration in the extraocular muscles [4]. Patients in the active stage have a good response to immunosuppressive treatment of glucocorticoid. Nevertheless, in the subsequently inactive phase, patients can no longer benefit from glucocorticoid treatment, with surgical decompression being the only solution [5, 6]. Thus, it is crucial in clinical practice to accurately diagnose the two stages and give the appropriate therapy.

Currently, we classify the two stages of TAO mainly using the Clinical Activity Score (CAS) [7]. However, it is unable to quantitatively evaluate individual muscle involvement using only this score. Orbit Magnetic resonance imaging (MRI) has been widely applied for diagnosing TAO and further staging TAO [8]. MRI provides the basis for the qualitative diagnosis of the pathologic changes in extraocular muscles. Nevertheless, quantitatively staging TAO using MRI remains limited. On the other hand, the conventional MRI scan is not ideal for discriminating fat from water-containing tissues, while the contrast-enhanced MRI is expensive and limited to be used in those with renal insufficiency or allergic reactions. The Short Tau (inversion time) Inversion Recovery (STIR) sequence of MRI has been reported to closely correlate with CAS [9, 10]. This sequence can selectively suppress signals from fat whilst highlighting water-containing tissues, thereby being more discerning in the inflammatory oedematous extraocular muscles [11]. Whether the signal intensity on STIR imaging can be applied to accurately evaluate the disease activity needs more clinical data. This prospective study aims to identify the cut-off signal intensity value for staging TAO using STIR sequence scan in Chinese patients.

## Methods

### Subjects

Between June 2018 and July 2020, a number of 51 patients with TAO who visited our hospital, and 31 volunteer controls, were consecutively recruited. All participants were between 18 and 65 years of age. The patients included met the TAO diagnostic criteria of Bartly [12]. The patients who had: (i) other causes of orbital disease such as tumor, trauma, or inflammation; and (ii) history of glucocorticoid therapy, radiotherapy, or surgical decompression surgery, were excluded. For the volunteer controls, the inclusion criteria include: (i) no history of thyroid disease; (ii) a normal thyroid function and serum levels of thyroid autoantibodies; and (iii) no orbital diseases. All subjects recruited signed the informed consent form and agreed to take part in the present study. The study was approved by the Ethical Committee of the First Affiliated Hospital of Chongqing Medical University.

### Clinical and biochemical measurements

Bodyweight and height were measured in light clothes and bare feet. Body mass index (BMI) was calculated as the ratio between weight in kilograms and the square of height in meters (kg/m^2^). Eyesight was checked using an international visual chart. Intraocular pressure (IOP) reading was measured with a noncontact tonometer (CT-1, Topcon, Japan). Proptosis was measured with a Hertel exophthalmometer (66vison Technology Co., Ltd., Suzhou, China) and recorded as millimeters. The free triiodothyronine (FT3), Free thyroxine (FT4), and thyroid stimulating hormone (TSH) were determined with the electro-chemiluminescence method (Unicel DxI 800 Immunoassay System, Beckman Coulter, USA). The thyroid peroxidase antibody (TPO-Ab), thyroglobulin (Tg-Ab), and thyroid stimulating receptor antibody (TR-Ab) were examined using chemiluminescence immunoassay (DXI800, Beckman, USA; Cobas601, Roche, Germany).

### Assessments of clinical activity score (CAS)

The assessment of CAS and severity for each eye was determined based on the guidelines of 2016ETA/EUGOGO [13], which was performed by an experienced endocrinologist on all patients. The CAS consists of seven findings: spontaneous retrobulbar pain; pain on attempted up or down gaze; redness of the conjunctiva; redness of the eyelids; inflammation of the caruncle and/or plica; swelling of the eyelids; and conjunctival edema. Each finding is scored 1 point. A summed CAS score of ≥3 is considered as an active TAO. Disease severity was divided into three grades: mild, moderate-severe, and very severe (Sight-threatening) based on having one or more of the following presentations: minor or sufficient impact on daily life, lid retraction≥2 mm, soft-tissue involvement, exophthalmos≥3 mm above normal, inconstant or constant diplopia, corneal exposure responsive to lubricants, dysthyroid optic neuropathy (DON) and/or corneal breakdown.

### Measurement of extraocular muscle signal intensity

Coronal orbital MRI was performed in each participant using a 3.0-T MRI scanner (Skyra, Siemens, Germany) with a 20-channel head coil. The signal intensities in the superior rectus, inferior rectus, lateral rectus, and medial rectus were measured on the STIR images (repetition time = 3700 ms; echo time = 48 ms; inversion time = 200 ms; slice thickness = 3 mm; matrix = 180X180; the number of excitations = 2). Five consecutive slices in each muscle were measured. The signal intensity of brain white matter (WM) on each slice was measured in order to normalize the signal intensity of muscles. The measurements were performed by two independent radiologists. The results of the two observers had excellent inter-reader agreement with an intraclass correlation coefficient (ICC) of 0.93. Then the results of the observer 1 were used for further statistical analysis.

### Statistical analysis

Statistical analysis was performed using SAS 9.13 (SAS Institute, Cary, NC). Variables were presented as means ± SD. Means of continuous variables between two groups were compared using the Unpaired t-test or Mann-Whitney Test (when the data were not normally distributed). The percentage differences between groups were compared using χ^2^ tests. The Logistic Regression analysis was performed to evaluate the associations between the signal intensity of extraocular muscles and disease activity. Receiver operating characteristic (ROC) curve analysis was performed to evaluate the predictive efficiency and the cut-off values for staging disease activity. Moreover, the accuracy of the identified cut-off values was further applied in a new group of TAO patients to determine the specificity and sensitivity for the cut-off values in the validation cohort. The intraclass correlation coefficient (ICC) was analyzed by Pearson correlation. The differences between groups were considered statistically significant at *P* < 0.05. For logistic regression analyses and ROC curve analysis, the statistical significance was set as P < 0.05.

## Results

### Clinical and biochemical characteristics of the active, inactive TAO patients and controls

All the TAO patients enrolled have not received the anti-TAO treatment including glucocorticoid therapy, radiotherapy, or surgical decompression surgery. Most patients had symmetrical activity of GO, except one had unilateral active GO. As shown in Table 1, compared with the control group, the overall TAO patients had significantly elevated serum levels of FT3 and thyroid autoantibodies (TPO-Ab, Tg-Ab, and TR-Ab), as well as abnormal ocular signs (increased intraocular pressure and degree of exophthalmos). Compared with the inactive TAO patients, the active TAO patients were older and consumed more tobacco. Also, the intraocular pressure and degree of exophthalmos were much worse in the active TAO patients. There were no significant differences in terms of sex, disease duration, BMI, thyroid function, and thyroid autoantibodies between the inactive and active groups.

**Table 1:** General Characteristics of subjects

	Control (*n* = 31)	TAO		*P*-Value		
		Inactive (*n* = 26)	Active (*n* = 25)	Inactive vs. Control	Active vs. Control	Active vs. Inactive
Eyes	62	51	51	–	–	–
Age (year)	46.10 ± 15.45	43.00 ± 12.93	51.56 ± 10.23	0.42	0.13	0.02
Male, n (%)	12 (38.71)	10 (38.46)	14 (56.00)	0.99	0.20	0.21
Duration (m)	–	6 (3–12)	5 (3–8)	–	–	0.24
Smoker, n (%)	4 (12.90)	7 (26.92)	14 (56.00)	0.18	< 0.01	0.02
BMI (kg/m^2^)	23.03 ± 2.92	22.96 ± 3.16	23.50 ± 2.62	0.93	0.53	0.51
FT3 (pg/ml)	2.94 ± 0.37	5.14 ± 4.48	3.53 ± 0.88	< 0.01	< 0.01	0.08
FT4 (ng/dl)	0.87 ± 0.10	2.17 ± 3.23	1.68 ± 2.64	0.03	0.09	0.56
TSH (μIU/ml)	1.64 (1.18–2.28)	0.15 (0.01–3.03)	0.50 (0.04–3.66)	0.44	0.20	0.75
TPO-Ab	0.60 (0.30–1.15)	44.00 (3.65–125.50)	11.00 (4.60–46.3)	< 0.01	0.01	0.98
Tg-Ab	0.10 (0.10–0.10)	0.80 (0.10–9.43)	0.20 (0.10–5.80)	< 0.01	0.03	0.57
TR-Ab	0.50 (0.35–0.50)	3.40 (0.90–20.35)	7.30 (3.48–18.70)	< 0.01	< 0.01	0.79
Eyesight	0.79 ± 0.25	0.82 ± 0.28	0.66 ± 0.28	0.50	< 0.01	< 0.01
Intraocular pressure (mmHg)	15.36 ± 3.02	17.53 ± 3.65	21.41 ± 4.87	< 0.01	< 0.01	< 0.01
Exophthalmos (mm)	15.66 ± 2.25	18.67 ± 3.62	20.31 ± 2.99	< 0.01	< 0.01	< 0.01
Disease severity						
mild, n (%)	–	18 (69.23)	1 (4.00)	–	–	< 0.01
moderate-severe, n (%)	–	8 (30.77)	17 (68.00)	–	–	< 0.01
very severe, n (%)	–	0	7 (28.00)	–	–	< 0.01

Table 1 Data are means ± SD or median (interquartile ranges) or number (percentage) for indicated number of patients in each group. *P* values for comparisons between groups are based on unpaired t test or Mann-Whitney Test or χ^2^ test.

*Abbreviations*: *BMI* Body mass index, *FT3* Free triiodothyronine, *FT4* Free thyroxine, *Tg-Ab* Thyroglobulin antibody, *TPO-Ab* Thyroid peroxidase antibody, *TR-Ab* Thyroid stimulating receptor antibody, *TSH* Thyroid stimulating hormone.

### Signal intensities of extraocular muscles in the inactive, active TAO patients and controls

**Fig. 1:**
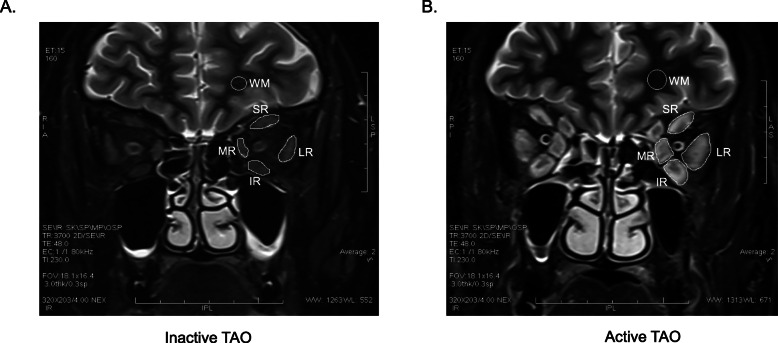
Measurement of signal intensities of extraocular muscles on STIR imaging in one inactive eye (**A**) and one active eye (**B**). The signal intensity in each muscle was normalized to that in white matter of the same slice. Abbreviations: SR = superior rectus; IR = inferior rectus; LR = lateral rectus; MR = medial rectus. WM = White matter

The signal intensities of the superior rectus, inferior rectus, lateral rectus, medial rectus, and superior oblique muscles on STIR images were measured in 51 active TAO eyes, 51 inactive TAO eyes, and 62 eyes from controls (Fig. 1). The values were divided by the signal intensity of brain white matter (WM) on the same slice, and data were presented as the signal intensity ratios (SIRs). The highest SIR value among the serial five slices (SIR-max) was adopted as the representative value of that muscle. The SIRs of four muscles in the active TAO group were significantly higher than those in the inactive TAO group and controls. The SIRs did not differ between the inactive TAO group and the controls (Table 2).

**Table 2:** The signal intensity ratios (SIRs) of extraocular muscles in the active thyroid-associated ophthalmopathy (TAO) group, inactive TAO group, and controls

	Control (*n* = 62 eyes)	TAO		*P*-Value		
		Inactive (*n* = 51 eyes)	Active (*n* = 51 eyes)	Inactive vs. Control	Active vs. Control	Active vs. Inactive
Superior rectus	2.12 ± 0.30	2.32 ± 0.55	3.12 ± 0.88	0.21	< 0.01	< 0.01
Inferior rectus	2.27 ± 0.46	2.63 ± 1.05	3.41 ± 0.81	0.42	< 0.01	< 0.01
Medial rectus	2.33 ± 0.48	2.43 ± 0.62	3.13 ± 0.81	0.36	< 0.01	< 0.01
Lateral rectus	2.28 ± 0.50	2.43 ± 0.65	2.87 ± 0.65	0.21	< 0.01	< 0.01

Data are means ± SD for indicated number of eyes in each group. *P* values for comparisons between groups are based on unpaired t test or Mann-Whitney Test

### Associations between extraocular muscle SIRs and CAS

The associations between the extraocular muscle SIRs and the disease activity were further analyzed. Considering that the age and proportion of smokers differed between the inactive and active TAO group, we performed multivariate logistic regression analyses to adjust these two confounders. When the four muscle SIRs were included individually in logistic regression analyses, they were closely associated with CAS after adjusting for age and tobacco usage (Table 3), indicating that the SIRs of extraocular muscles were strong predictors for the activity of TAO (Table 3).

**Table 3:** Associations between the signal intensity ratios (SIRs) of extraocular muscles and the disease activity of Thyroid-associated ophthalmopathy (TAO)

	OR (95% CI)	*P*-value
Adjusted for age and smoker		
Superior rectus	3.76 (1.86–7.60)	< 0.01
Inferior rectus	1.91 (1.17–3.12)	< 0.01
Medial rectus	2.93 (1.43–6.01)	< 0.01
Lateral rectus	2.69 (1.24–5.85)	0.01

The Clinical Activity Score (CAS) was treated as dichotomous variables.

*Abbreviations*: *OR* odds ratio, *CI* confidence interval

### Receiver operating characteristic curve analysis

To determine the predictive efficiency and diagnostic cut-off values that are predictive of the active stage, we then performed receiver operator characteristic (ROC) curve analysis. All four muscle SIRs demonstrated optimal efficiency for prediction [area under curve (AUC) 0.76–0.83, all *P* < 0.01] (Table 4). When comparison of the four ROC curves, the AUCs among the superior rectus, inferior rectus, and medial rectus were not significantly different. The AUC of the lateral rectus was almost significantly lower than that of the inferior rectus (Difference between areas:0.068, *P* = 0.05). The detailed cut-off SIR values, specificity, and sensitivity were displayed in Table 4. Considering that there were no histopathological results to confirm the exact phases of muscles in our study, the diagnostic accuracy of the identified cut-off values was further validated in another fifteen TAO patients (30 eyes) enrolled between September 2020 and January 2021(for characteristics of the patients see Supplementary Table). These patients were staged according to the identified cut-offs and matched with their actual CAS. The fit model showed the highest specificity and sensitivity for the cut-off value of the inferior rectus (cut-off value 2.90, specificity 100%, sensitivity 93.75%) (Table 4), suggesting a robust classification power of this muscle.

**Table 4:** Predictive performance of signal intensity ratios (SIRs) of extraocular muscles between the active and inactive Thyroid-associated ophthalmopathy (TAO)

	AUC	Cut-off	Specificity%	Sensitivity%	*P*-value	Validation of Cut-offs	
						Specificity%	Sensitivity%
Superior rectus	0.83	2.48	80.50	74.50	< 0.01	91.67	81.25
Inferior rectus	0.83	2.90	84.07	76.47	< 0.01	100.00	93.75
Medial rectus	0.80	2.57	77.00	70.60	< 0.01	83.33	87.50
Lateral rectus	0.76	2.74	83.20	60.80	< 0.01	91.67	75.00

The Clinical Activity Score (CAS) was treated as dichotomous variables.

*Abbreviation*: *AUC* area under curve

## Discussion

In the present study, we aimed to establish a cut-off SIR value of extraocular muscles for staging TAO. We took advantage of the STIR sequence of MRI to scan the extraocular muscles because the high signal intensity indicates edema caused by inflammation in extraocular muscles. The use of STIR scans in TAO patients has previously been established. Several studies in European patients observed that changes in signal intensity of the extraocular muscles on STIR closely correlate with CAS [9, 10, 14, 15], demonstrating that the STIR sequence was a valuable tool for evaluating disease activity. Whereas rare studies have established the precise signal intensity values for classifying disease activity. Our results further proved the significant increased extraocular muscle SIRs in the active TAO and the strong association between SIRs and disease activity, confirming the potency of STIR technique in characterizing inflammatory changes of orbital tissues in Chinese patients. Moreover, by ROC analysis, we found that all four extraocular muscles had good performance in predicting the active stage of TAO. The SIR in the inferior rectus had the best predicting efficiency with the highest specificity and sensitivity. After validating the cut-off values in a new group of TAO patients, we found that the cut-off value of 2.9 in the inferior rectus had optimal specificity and sensitivity. Thus, we proposed a cut-off value of > 2.9 in inferior rectus on STIR for diagnosing the active stage of TAO. In anatomical and histologic findings, the inferior rectus has been shown as the most commonly inflamed muscle among the extraocular muscles [2, 16–18]. Some studies showed that less expressed collagen XIII alpha 1 (known to be essential to the development and maintenance of neuromuscular junctions and help support normal immune function) in the inferior rectus might explain the vulnerability of this muscle [19]. Therefore, the cut-off SIR value of the inferior rectus could be the preferred one for assessing the stage of TAO in clinical practice.

It should be mentioned that due to the difficulties in achieving the histopathological analysis of extraocular muscles, the exact phase of muscles is often unclear. Although the identified cut-offs were validated in a new group of patients, the sample used for validation was relatively small. The accuracy of the predicting values needs to be validated in a future large population. Also of note, although the prevalence of TAO is higher in women than in men, the proportions of female and male patients were similar in our study. We speculate that this discrepancy may result from the selection bias. Studies have demonstrated that this disease tends to be relatively more severe in men [4, 20]. Thus, a greater number of men may go to hospital and therefore be recruited, which may alter the female-to-male ratio.

In conclusion, our study showed that the signal intensity of extraocular muscles on the STIR sequence was a good predictor for TAO activity. A cut-off SIR value of > 2.9 in the inferior rectus could be preferably to evaluate the active stage of TAO. Using the SIR values obtained with the STIR sequence may help to further quantitatively stage TAO and determine the appropriate treatment. Our findings in the Chinese patient population may or may not be generalizable to the world at large.

## Supplementary Information

**Additional file 1.**

## Data Availability

The datasets generated during and/or analyzed during the current study are available from the corresponding author on reasonable request.

## Abbreviations

AUC: Area under curve
BMI: Body mass index
CAS: Clinical activity score
FT3: Free triiodothyronine
FT4: Free thyroxine
MRI: Magnetic resonance imaging
TAO: Thyroid-associated ophthalmopathy
Tg-Ab: Thyroglobulin antibody
TPO-Ab: Thyroid peroxidase antibody
TR-Ab: Thyroid stimulating receptor antibody
TSH: Thyroid stimulating hormone
SIR: Signal intensity ratio
STIR: Short Tau Inversion Recovery
