# Supplementary material for: Quantitative evaluation of activity of thyroid-associated Ophthalmopathy using short-tau inversion recovery (STIR) sequence
Source: BMC Endocr Disord. 2021 Nov 13;21:226. doi: 10.1186/s12902-021-00895-3 (PMC8590769; doi:10.1186/s12902-021-00895-3)
Supplement: Supplementary file 1 — Additional file 1. [file 12902_2021_895_MOESM1_ESM.docx]

Supplementary Table. The characteristics of the second recruited patients with Thyroid-associated Ophthalmopathy (TAO)

|  | TAO patients (n=15) |
| --- | --- |
| Eyes | 30 |
| Age (year) | 49.21±11.04 |
| Male, n (%) | 14 (26.67) |
| Duration (m) | 6.00(3.25-16.00) |
| Smoker, n (%) | 4 (12.90) |
| BMI (kg/m^2^) | 23.30±2.19 |
| FT3 (pg/ml) | 3.08(2.91-3.59) |
| FT4 (ng/dl) | 0.94(0.65-1.07) |
| TSH (μIU/ml) | 0.07(0.01-2.76) |
| TPO-Ab | 89.50(14.03-391.23) |
| Tg-Ab | 10.00(0.13-27.04) |
| TR-Ab | 6.05(2.25-17.55) |
| Eyesight | 0.68±0.29 |
| Intraocular pressure  (mmHg) | 20.21±4.51 |
| Exophthalmos  (mm) | 19.36±3.05 |
| CAS | 3 (1.25-4) |
| Inactive, n (%) | 6 (40) |
| Active, n (%) | 9 (60) |
| Disease severity |  |
| Mild, n (%) | 4 (26.67) |
| Moderate-severe, n (%) | 8 (73.33) |
| Very severe, n (%) | 3 (20) |

These patients were enrolled between September 2020 and January 2021, in order to validate the accuracy of the identified muscle SIR cut-offs. Data are means ± SD or median (interquartile ranges) or number (percentage). Abbreviations: BMI=Body mass index, CAS: Clinical Activity Score, FT3=Free triiodothyronine, FT4=Free thyroxine, Tg-Ab=Thyroglobulin antibody, TPO-Ab=Thyroid peroxidase antibody, TR-Ab=Thyroid stimulating receptor antibody, TSH=Thyroid stimulating hormone. SIR: signal intensity ratio.
